# Facial and dental arch shape in individuals with different bite force levels: A pilot study using geometric morphometrics

**DOI:** 10.1007/s00056-024-00553-y

**Published:** 2024-10-28

**Authors:** Ana Nocera Quezada, María Jesús Muñoz, Ellen Schulz-Kornas, Maximilian Bemmann, Kornelius Kupczik, Oliver Schierz, Viviana Toro-Ibacache

**Affiliations:** 1 Laboratory of Craniofacial Translational Research, Faculty of Dentistry, University of Chile, Olivos 943, Independencia, Santiago, Chile; 2 Department of Cariology, Endodontics and Periodontology, University of Leipzig, Liebigstraße 12, 04103 Leipzig, Germany; 3 Max Planck Weizmann Center for Integrative Archaeology and Anthropology, Deutscher Platz 6, 04103 Leipzig, Germany; 4 Department of Anthropology, Faculty of Social Sciences, University of Chile, Capitán Ignacio Carrera Pinto 1045, Ñuñoa, Santiago, Chile; 5 Department of Prosthetic Dentistry and Materials Science, University of Rostock, Strempelstraße 13, 18057 Rostock, Germany

**Keywords:** Geometric morphometrics, Facial morphology, Facial shape, Human adults, Pilot project, Geometrische Morphometrien, Morphologie des Gesichts, Gesichtsform, Erwachsene Menschen, Pilotprojekt

## Abstract

**Purpose:**

This pilot study aimed to assess the relationship between bite force variation and dental arch and facial shape using geometric morphometrics, an advanced method of statistical analysis that provides a detailed shape analysis of a structure considering the spatial relationship of its parts.

**Methods:**

The sample consisted of 16 German adult men and women. For each individual, maximum bite force was recorded in four positions: maximum intercuspation, protrusion, laterotrusion to the right and to the left. Facial and three-dimensional (3D) dental reconstructions were obtained from 3D facial photographs and 3D scans of dental stone models. A total of 51 landmarks were placed. General shape variation was assessed by principal component analysis. Partial least squares analyses were performed to evaluate the covariation between bite force, facial shape, and dental shape.

**Results:**

There was no clear pattern or statistically significant covariation between our variables.

**Conclusions:**

Our results suggest a weak relationship between bite force, dental arch, and facial shape. Considering previous work in this field, we propose that low masticatory loads, characteristic in Western urban populations, may explain this. Further studies should, therefore, address this issue, taking into account effect size, the mechanical properties of the diet, and other relevant variables.

## Abstract (de)

**Ziel:**

Ziel dieser Pilotstudie war es, die Beziehung zwischen Okklusionskraftvariation sowie Zahnbogen- und Gesichtsform mit Hilfe der geometrischen Morphometrie zu untersuchen. Die geometrische Morphometrie ist eine fortgeschrittene statistische Analysemethode, die eine detaillierte Formanalyse einer Struktur unter Berücksichtigung der räumlichen Beziehung ihrer Teile ermöglicht.

**Methoden:**

Die Stichprobe bestand aus 16 erwachsenen deutschen Männern und Frauen. Für jede Person wurde die maximale Okklusionskraft in 4 Positionen aufgezeichnet: maximale Interkuspidation, Protrusion, Laterotrusion rechts und links. Gesichts- und dreidimensionale Zahnrekonstruktionen wurden anhand von 3D-Fotos des Gesichts und 3D-Scans von Modellen erstellt. Insgesamt wurden 51 Referenzpunkte festgelegt. Die allgemeine Formvariation wurde mittels Hauptkomponentenanalyse ausgewertet. PLS(„Partial Least Squares“)-Analysen wurden durchgeführt, um die Kovariation zwischen Okklusionskraft, Gesichtsform und Zahnform zu bewerten.

**Ergebnisse:**

Es gab kein klares Muster oder eine statistisch signifikante Kovariation zwischen unseren Variablen.

**Schlussfolgerung:**

Unsere Ergebnisse deuten auf einen schwachen Zusammenhang zwischen Bisskraft, Zahnbogen und Gesichtsform hin. Unter Berücksichtigung früherer Arbeiten auf diesem Gebiet vermuten wir, dass sich dies durch die für westliche Stadtbevölkerungen charakteristische geringe Kaubelastung erklären könnte. Weitere Studien sollten sich daher mit dieser Frage befassen und dabei die Effektgröße, die mechanischen Eigenschaften der Nahrung und andere Faktoren berücksichtigen.

## Introduction

Human muscles and bones are developmentally related through their anatomy and molecular physiology [1]. The forces exerted by the muscles affect bone morphology through the process of bone modeling and remodeling. When muscles contract, they induce microdeformations in the bone in which they are inserted, as well as in the bones loaded by the action of the muscle contraction. Increasing or decreasing these strains causes changes in the amount of bone being formed and reabsorbed, as well as modulating the direction of its growth [2]. It has been proposed that in modern Western populations, due to their diets based on highly processed foods, less chewing is required, and thus less bone deformation is induced by the chewing muscles, resulting in less developed bony structures of the skull and face [3].

The muscle–bone relationship is of clinical importance in dentistry, and the craniofacial complex can be evaluated from a morphological or functional perspective. In this regard, analyzing the shape of the face and the dental arch shape is relevant as a morphological variable. In contrast, bite force (BF) is an indicator of the function of the masticatory system.

As assessed by either cephalometric or anthropometric methods, there are three patterns traditionally used to describe facial shape based on their width–length ratio: long (dolichofacial), wide (brachyfacial), and average faces (mesofacial) [4, 5]. Yet, there is less consensus on the classification of dental arches shape. The relationship between face and dental arch shape has mainly been studied using linear or angular measurements. Long faces have been associated with a narrow dental arch, whereas short face patterns would show a comparatively wider arch [6–8]. Average faces fall somewhere in between. However, some studies failed to find a significant relationship between facial and dental arch shape [9]. The degree of covariation between two or more anatomical elements, known as morphological integration, implies that coordinated patterns of shape variation are the result of developmental, evolutionary, genetic, and functional processes. In contrast, the anatomical independence of the craniofacial elements is defined as modularity [10]. However, whether the face and the dental arch in humans show integration or modularity, despite their anatomical and functional relationship, is not entirely clear.

Bite force is one of the most defining functional variables of the masticatory system. The chewing process generates loads that are important for shaping bone during craniofacial development [1, 11]. Hence, it should be expected that BF has a direct effect on the shape of the adult face and dental arch. To date, this relationship has been studied through clinical examination, photographs, cephalometric analysis, and morphometrics of dental models [4, 5, 12, 13]. Variations in BF and their underlying factors have been commonly evaluated by direct BF measurements, electromyographic measurements, and anatomical studies of the masticatory muscles [14–16]. The relationship with face shape has been extensively studied, using linear or angular measurements [17–20]. Most studies claim that broad faces have the highest BF magnitudes, while long faces have the lowest [17, 18]. Regarding the relationship between BF and dental arch shape, it should be noted that the relative position and length of the dental arch may influence the stress and strain distribution on the skull [2, 21]. However, studies in capuchin monkeys [22] and modern humans [23] have shown that the shape (as assessed by geometric morphometric methods) of the dental arch, as opposed to size and position, is independent of BF. Despite this information, the evolutionary and biomechanical mechanisms underlying the shape of the dental arch between humans and apes are not well understood [24]. From a mechanical perspective, the question arises as to whether there is a relationship between facial morphology, dental arch, and function (BF). The present study addressed this question using three-dimensional landmark-based geometric morphometrics (GM). Unlike traditional shape evaluation methods, GM is an advanced statistical method that provides a detailed shape analysis, taking into account the geometry and spatial relationship of the analyzed object rather than being limited to the analysis of its size or to finite categories. Thus, shape is treated as a continuous variable. The raw data representing the object geometry are landmarks, which are points in space whose Cartesian coordinates indicate their location in a two- or three-dimensional space, and have a correspondence between individuals in terms of biological homology [25, 26]. The GM method consists of three main steps: (1) obtaining the raw data (representative landmarks of the shape to be studied), (2) obtaining information about the pure *shape* by eliminating from the original, raw data, the differences in geometry that may be due to the size, position, and rotation of the object, and (3) exploratory and confirmatory analysis of the covariation of the shape (i.e., the “new” coordinates) and its underlying factors [25, 26]. Because GM evaluates the covariation between the shape of a structure and its underlying factors (e.g., diagnose, physiological parameters), studies using these methods have great potential for studying patients with morphofunctional alterations of the stomatognathic system. Thus, this method is being increasingly used in dentistry, specifically in geriatric dentistry and oral rehabilitation [27], maxillofacial surgery [28–30], orthodontics [31, 32], among others. Since there currently is no clear evidence regarding the anatomical and functional relationship between FS, DAS, and BF, we explore here the use of a new methodological framework that incorporates shape and functional data, obtained with systems that are increasingly available among clinicians to address this question. Specifically, this pilot study aims to test the null hypothesis that there is no correlation between the shape of the face (FS), the dental arch (DAS), and the BF in the sample using GM tools.

## Materials and methods

Ethical approval for the use of image data and BF measurements was obtained from the Ethics Committee of the Faculty of Medicine, University of Leipzig (No. 396-15-13072015, Leipzig, Germany). Data processing and analyses were performed at the Max Planck Institute for Evolutionary Anthropology (Leipzig, Germany) and the Faculty of Dentistry of the University of Chile (Santiago, Chile). The trial is registered in the German Clinical Trials Register under DRKS00009787.

The sample of this pilot study consisted of 16 German individuals (6 men, 10 women) who met the following criteria: 23–30 years of age, body mass index of 20–24.9 for men and 19–23.9 for women, full dentition (without considering third molars) and absence of muscular and/or temporomandibular joint symptoms. Individuals with extreme alterations in occlusion (overjet >5 mm, overbite >5 mm, crossbite and/or open bite), history of orthognathic surgery or orthodontic treatment, history or presence for treatment of temporomandibular disorders, muscular and/or temporomandibular joint functional pathology, orofacial pain, history or presence of periodontitis, and dental restorations that prevent the recognition of landmarks were excluded. We did not use narrow inclusion criteria, e.g., occlusion type, in order to allow for a wide (but not extreme) morphologic variation within the sample.

### Bite force data

BF data were obtained with the BiteFork System (ViMeS, Igel, Germany), which measures force intraorally through 0.2-mm-thick piezosensitive sensors (Tekscan, Norwood, MA, USA) between a 2–5 mm high bite block. The system digitizes the measured voltage converting it to newton units (N) [33]. Tekscan sensors are widely and reliably used for in vivo bite force measurements [34, 35].

Maximum BF was recorded at different positions: maximum intercuspation (MIC), protrusion, and laterotrusion to the left and right. Forces at the MIC and lateral occlusion were measured at the first molar, whereas for protrusion they were measured between the central incisors. A custom-made silicone fixator was placed mesially of the first molar (MIC and lateral occlusion) and between the central incisors (protrusion) for repeatability purposes. Since there was a sensor on each side to measure the BF at MIC, two results were obtained: right MIC and left MIC. The average of the two (average MIC) was calculated. Overall, there were 6 force values or positions studied: right MIC, left MIC, average MIC, protrusion, left laterotrusion, and right laterotrusion.

### Shape data

**Fig. 1:**
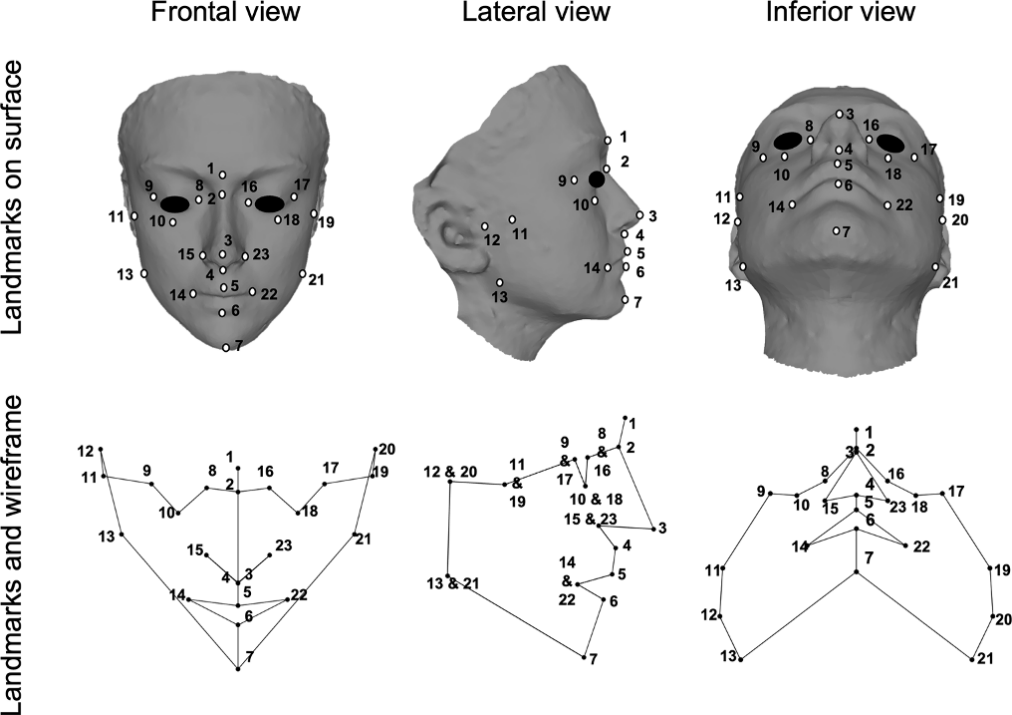
Selected facial landmarks and the wireframes obtained on MorphoJ Ausgewählte Referenzpunkte im Gesicht und mit MorphoJ erstellte Netzgitter

Three-dimensional reconstructions of the face and dental arches were obtained from 3D facial photographs by stereophotogrammetry using the Vectra M3 facial scanner and its associated software (Canfield, Parsippany, NJ, USA) by a single observer (O.S). Prior to taking the 3D photographs, an orthodontist marked landmarks on the participant’s skin with a pen after palpation. The resulting wavefront texture files (.obj format) were used to analyze the facial anatomy. These files were imported into MeshLab software (v.1.3.3, ISTI-CNR, Pisa, Italy) [36], and the 3D coordinates of each landmark were digitized. The list of 23 landmarks was selected according to Mizayato et al. [37] and is presented in Table 1 and Fig. 1.

**Table 1:** Selected facial landmarks Ausgewählte Referenzpunkte im Gesicht

Structure	Abb.	Landmark	Definition	M‑R/L	No.
Forehead	G	Glabella	Most prominent point located on the forehead in the midsagittal plane, between the supraciliary arches [38]	M	1
	Se	Sellion	Point located in the intersection between the nasofrontal suture and the midsagittal plane [38]. In soft tissue, it represents the most concave point that covers the area of the frontonasal suture [39]	M	2
Eyes/Orbit	En	Entocanthion	A point located in the mesial commissure of the orbital cavity [40, 41]	R/L	8/16
	Ex	Exocanthion	A point located in the lateral commissure of the orbital cavity [40, 41]	R/L	9/17
	Inf	Infraorbitale	Lowest point on the lower margin of each orbit [39]	R/L	10/18
Nose	Prn	Pronasale	Most anterior point of the nasal tip [42, 43]	M	3
	Sn	Subnasale	Point in the midsagittal plane where the nasal base meets the upper lip [43], being the midpoint at the angle of the base of the columella [41]	M	4
	Ac	Alar curvature	Most lateral point of the alar curvature [44]	R/L	15/23
Cheeks	Zy	Zygion	Most lateral point of the zygomatic arch [38]	R/L	11/19
	Cdl	Condylion laterale	Most lateral point on the mandibular condyles [45]	R/L	12/20
Mouth	Chl	Chellion	Lateral point located at each labial commissure [41]	R/L	14/22
	Ls	Labiale superius	Midpoint located on the vermilion border of the upper lip [40–42]	M	5
	Li	Labiale inferius	Midpoint located on the vermilion border of the lower lip [40–42]	M	6
Mandible	Sgn	Supragnathion	Midpoint located in the most anteroinferior position of the chin, between the pogonion and gnathion [27]	M	7
	Go	Gonion	Lateral point where the lower margin of the mandibular body meets the posterior margin of the ramus, being the point of the mandibular angle in its most inferior, posterior, and lateral position [38]	R/L	13/21

*Abb.* Abbreviation of the landmark; *M* Median; *R* Right; *L* Left; *No.* Number of the landmark

**Fig. 2:**
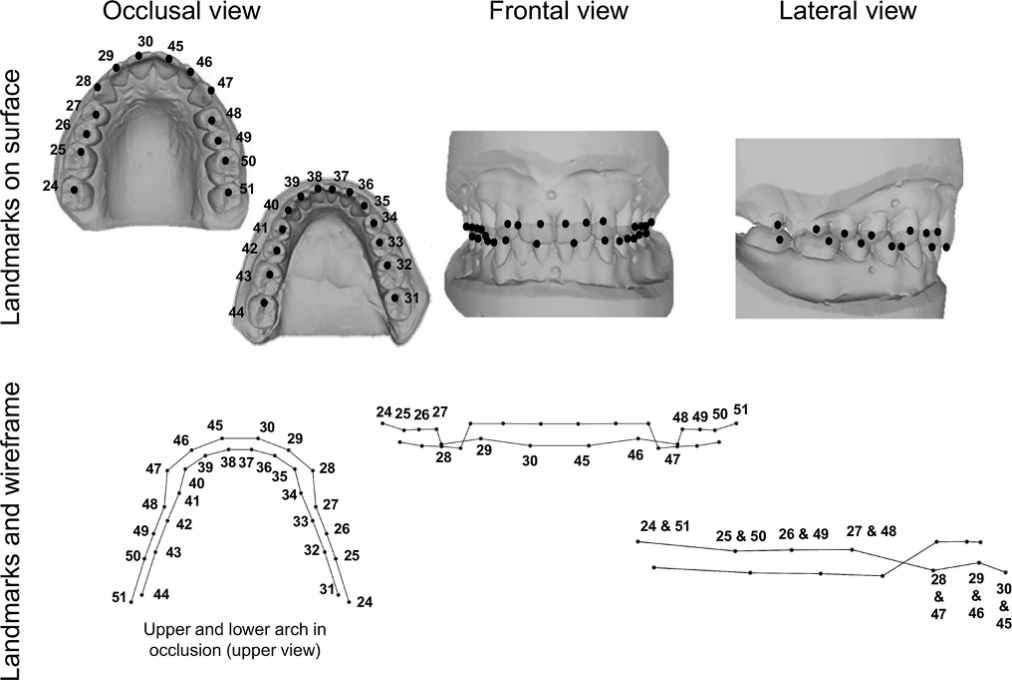
Selected dental arch landmarks and the wireframes obtained on MorphoJ. In the frontal and lateral views, only the maxillary dental arch landmarks are shown as a reference Ausgewählte Referenzpunkte im Gesicht und die mit MorphoJ erstellten Netzgitter. In der Frontal- und Seitenansicht sind nur die Referenzpunkte am Oberkieferzahnbogen zur Orientierung eingezeichnet

Stone models of the maxillary and mandibular arches were obtained and scanned separately using the Organical Scan D250 and its associated software (3Shape, Copenhagen, Denmark), resulting in three-dimensional reconstructions as stereolithography (.stl) files. In the Avizo v9.1 software (Science Visualization Group, Burlington, MA, USA), 28 landmarks representing the DAS were placed on the three-dimensional reconstructions (Table 2 and Fig. 2).

**Table 2:** Selected dental arch landmarks Ausgewählte Referenzpunkte im Zahnbogen

Structure	Abb.	Landmark	Definition	R/L	No.
Maxillary dental arch	M2S	Second molar	A point located in the center of the mesial fossa of the upper second molar	R/L	24/51
	M1S	First molar	A point located in the center of the mesial fossa of the upper first molar	R/L	25/50
	PM2S	Second premolar	A point located in the center of the main sulcus of the upper second premolar	R/L	26/49
	PM1S	First premolar	A point located in the center of the main sulcus of the upper first premolar	R/L	27/48
	CS	Canine	A point located in the center of the cusp of the upper canine	R/L	28/47
	ILS	Lateral incisor	A point located in the middle of the incisal edge of the upper lateral incisor	R/L	29/46
	ICS	Central incisor	A point located in the middle of the incisal edge of the upper central incisor	R/L	30/45
Mandibular dental arch	M2I	Second molar	A point located in the center of the mesial fossa of the lower second molar	R/L	31/44
	M1I	First molar	A point located in the center of the mesial fossa of the lower first molar	R/L	32/43
	PM2I	Second premolar	A point located in the center of the mesial fossa of the lower second premolar	R/L	33/42
	PM1I	First premolar	A point located in the center of the mesial fossa of the lower first premolar	R/L	34/41
	CI	Canine	A point located in the center of the cusp of the lower canine	R/L	35/40

*Abb.* Abbreviation of the landmark; *R* Right; *L* Left; *No.* Number of the landmark

Landmarks representing the shape of the face and dental arch were selected according to the Bookstein, Dryden, and Mardia criteria for biological landmark data [46, 47]. The landmarks were marked by the same observer on two different occasions and later digitized in Morphologika format (A.N.Q. for facial landmarks, M.M.R. for dental arch landmarks). This resulted in three datasets per individual for subsequent analyses: BF data, FS and DS landmark coordinates. The latter were exported as text files for use in subsequent analyses.

### Statistical analyses

#### Bite force

Using PAST3 software [48], a preliminary permutational multivariate analysis of variance (PERMANOVA; [49]) was performed to assess whether sex influenced BF overall. Subsequently, a Kruskal–Wallis test was performed for each bite position. Finally, a principal component analysis (PCA) was performed to assess which BF position was most relevant in the variation of the data.

#### Shape

In MorphoJ v.1.07 [38], a geometric morphometric analysis was performed on the shape variables obtained by Procrustes fit, consisting of translation, rotation, and scaling, which standardized the size and geometry of the raw landmark configurations. As a result, a reference, average shape, was obtained to quantify the shape changes of the subjects in the sample, known as the consensus configuration [25]. Wireframes were used to visualize and describe the variations in FS and DAS with respect to the consensus configuration (Figs. 1 and 2). These new landmark configurations (or “shape variables”), after Procrustes fit, are now suitable for the upcoming multivariate statistical analysis. As this is a pilot study, our study focuses on the descriptive aspect of shape variation.

The intraobserver error of the landmarks was assessed with an analysis of variance specific to shape variables (Procrustes ANOVA). This analysis was also performed to evaluate the effect of sex on shape variation. In this sample, there was a statistically significant effect of sex on FS variation but not on DAS variation. Therefore, a discriminant function analysis (DFA) was performed to determine if there was a significant difference between the two sexes. Finally, sex was used as a dummy variable (female = 0, male = 1) in a regression of FS variables against sex, to obtain residuals (shape variables free of sexual dimorphism) [39].

Due to the small sample size, statistical significance was evaluated using permutation tests (10,000 permutation rounds). For both preliminary and subsequent analyses, the statistical significance level was set at *p* < 0.05.

As the human face can show subtle asymmetries that are not of interest to this study, a data symmetrization process was performed to eliminate asymmetry, which acts as a confounding variable. Thus, the symmetric components of DAS and the residuals of sex regression of FS were used in the following analyses.

The general shape variation of the symmetrized FS and DAS was assessed using PCA. For geometrical data such as shape variables, the information provided by the principal components analysis is somewhat different from those analyses based on linear metrics. Each principal component (PC) shows combinations of geometric features that are relevant (decreasingly) in the observed variation of the shape among individuals in the sample. This information can, therefore, be described and results be interpreted in relation to the independent variables under study. For this study, the principal components (PC) that together explain most of the shape variation were analyzed. The descriptions of the shape variations represented by each PC were visualized through wireframes in the three axes of space related to the consensus configuration. Finally, three partial least squares (PLS) analyses (FS vs. DAS; FS vs. BF; DAS vs. BF) were performed to find and quantify a covariation between the studied variables.

## Results

### Bite force

The highest BF recorded in this sample was recorded by a male individual in right lateral occlusion (649 N), and the lowest by a female individual in protrusion (28 N). However, PERMANOVA (*p* = 0.058) and Kruskal–Wallis test showed no effect of sex on BF (all *p* > 0.05; Table 3).

**Table 3:** Bite force (BF) magnitudes and the effect of sex Ausmaß der Bisskraft (BF) und Einfluss des Geschlechts

Position	Median in newton (min./max. values)		Kruskal–Wallis
	Men	Women	*p*-value
Left MIC	307.381 (249.776/399.819)	250.029 (99.586/406.964)	0.100
Right MIC	136.779 (64.252/515.453)	137.099 (48.561/283.403)	1.000
Average MIC	268.171 (167.397/418.421)	206.677 (104.239/292.761)	0.190
Protrusion	117.664 (81.372/301.032)	76.628 (27.853/273.039)	0.150
Right laterality	131.576 (59.231/648.929)	246.038 (78.912/406.497)	0.440
Left laterality	397.838 (330.313/496.461)	339.390 (106.282/627.615)	0.190

*MIC* maximum intercuspation, *Min*. minimum, *Max*. maximum

Results for PCA showed that for PC1 (60.5%) that the three most relevant positions for BF were average MIC, right MIC, and right lateral occlusion.

### Shape data

ANOVA for intraobserver error of landmark configurations was set to *p* < 0.05. This means that the interindividual variation of the sample is significantly greater than the intraindividual variation derived from repeated landmarking [39].

ANOVA and DFA for sex showed that there was a statistically significant effect of sex on FS variation (*p* < 0.005), but not on DAS variation (*p* > 0.05).

#### Facial shape

**Fig. 3:**
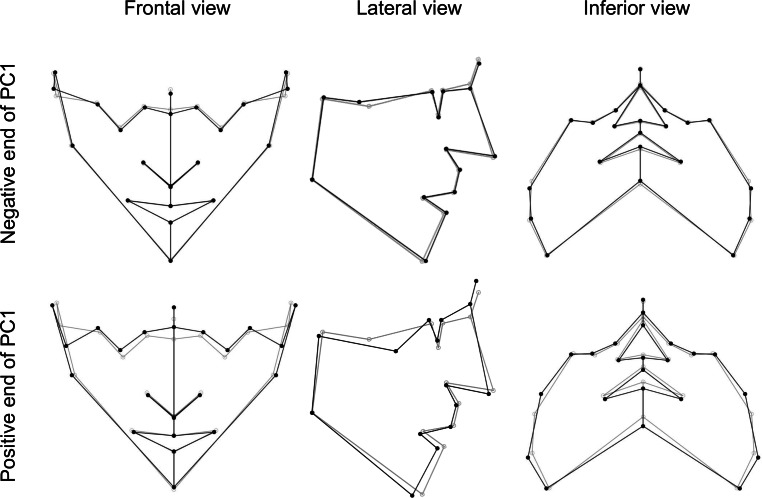
Visualization of face shape changes in first principal component (PC1). The *gray *wireframe corresponds to the reference, consensus configuration Visualisierung von Veränderungen der Gesichtsform in der ersten Hauptkomponente (PC1). Das *graue* Netzgitter entspricht der Referenz, Konsenskonfiguration

The first three principal components described 58.7% of the variation of the FS in the sample. The following descriptions were compared to the consensus FS, and visible in Fig. 3.

The first principal component (PC1) explained 27.7% of the total variation in FS in this sample. Individuals at the positive end of PC1 showed an increased anterior and decreased posterior facial height, with a mandibular angle higher than consensus. Anterior landmarks are slightly retruded in the sagittal view, and zygion was the landmark with the most variation (in an anterior and lower direction). Individuals at the negative end of PC1 showed a slight decrease in anterior facial height, a slight increase in posterior facial height, a lower mandibular angle, mild protrusion of anterior landmarks in a sagittal view, and zygion was the landmark with the most variation (posterior and superior; Fig. 3).

**Fig. 4:**
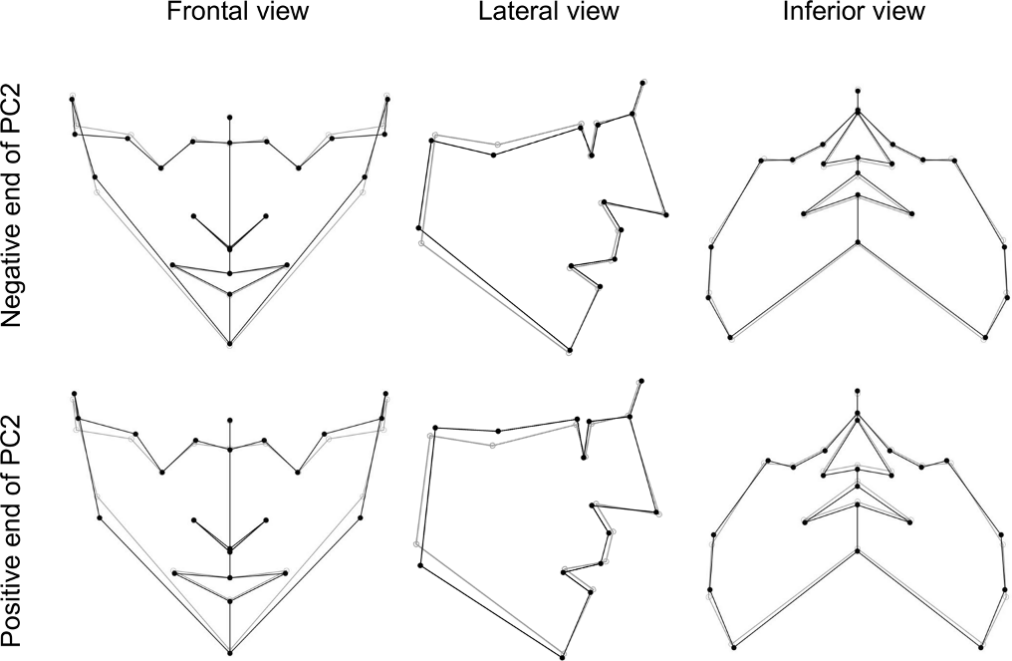
Visualization of face shape changes in second principal component (PC2). The *gray* wireframe corresponds to the reference, consensus configuration Visualisierung von Veränderungen der Gesichtsform in der zweiten Hauptkomponente (PC2). Das *graue* Netzgitter entspricht der Referenz, Konsenskonfiguration

The second principal component (PC2) explained 19.1% of the total variation of FS in this sample. This varied from an FS with both facial heights increased and a lower mandibular angle to an FS with both facial heights decreased and a higher mandibular angle (Fig. 4).

**Fig. 5:**
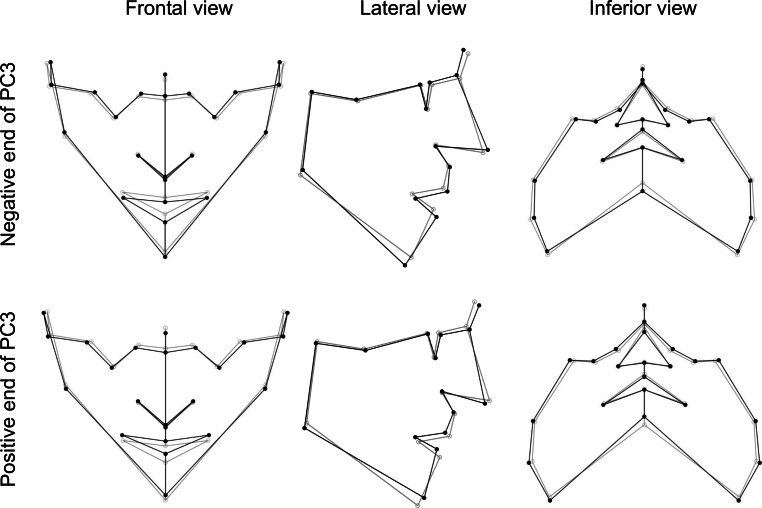
Visualization of face shape changes in third principal component (PC3). The *gray* wireframe corresponds to the reference, consensus configuration Visualisierung von Veränderungen der Gesichtsform in der dritten Hauptkomponente (PC3). Das *graue* Netzgitter entspricht der Referenz, Konsenskonfiguration

The third principal component explained 11.8% of the variation in FS in this sample. It varied from an FS with anterior facial height increase, posterior facial height decrease, and higher mandibular angle, suggesting a posterior mandibular rotation to an FS with an anterior facial height decrease, posterior facial height increase, and lower mandibular angle, suggesting an anterior mandibular rotation (Fig. 5).

#### Dental arch shape

The first three principal components described 68.3% of the variation in DAS in the sample. The following descriptions are compared to the consensus DAS.

**Fig. 6:**
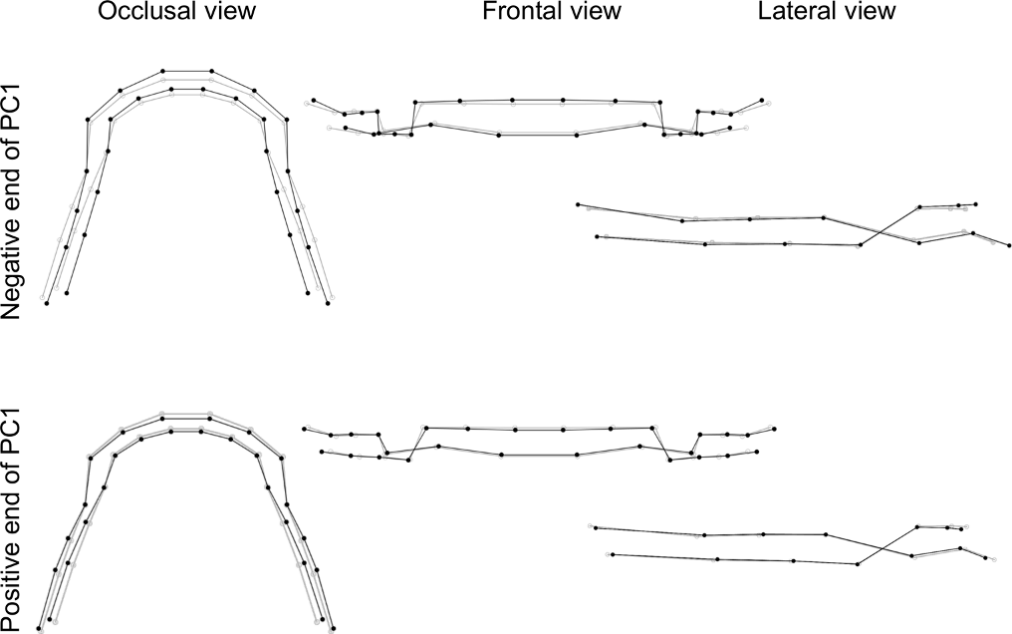
Visualization of dental arch shape changes in first principal component (PC1). The *gray* wireframe corresponds to the reference, consensus configuration Visualisierung von Veränderungen des Zahnbogens in der ersten Hauptkomponente (PC1). Das *graue* Netzgitter entspricht der Referenz, Konsenskonfiguration

The first principal component explained 32.6% of the variation in DAS in this sample. This varied from a DAS with posterior teeth positioned more lingually and distally and anterior teeth protruded (suggesting an enlarged, parabolic, and compressed DAS), to a DAS with posterior teeth positioned more vestibularly and mesially and anterior teeth retruded (suggesting a wider transverse arch and a shorter anteroposterior arch; Fig. 6).

**Fig. 7:**
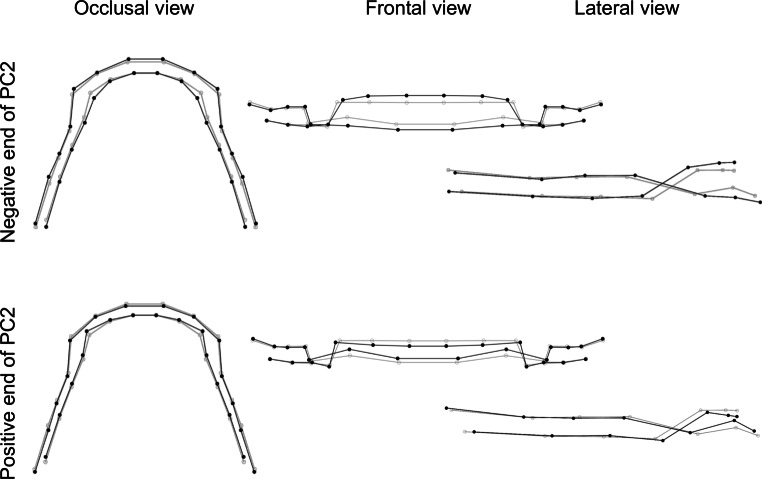
Visualization of dental arch shape changes in second principal component (PC2). The *gray* wireframe corresponds to the reference, consensus configuration Visualisierung von Veränderungen des Zahnbogens in der zweiten Hauptkomponente (PC2). Das *graue* Netzgitter entspricht der Referenz, Konsenskonfiguration

The second principal component explained 24.6% of the variation in DAS in this sample. Focusing on the frontal view, the DAS varied from a marked overbite to a DAS with a mild overbite (Fig. 7).

**Fig. 8:**
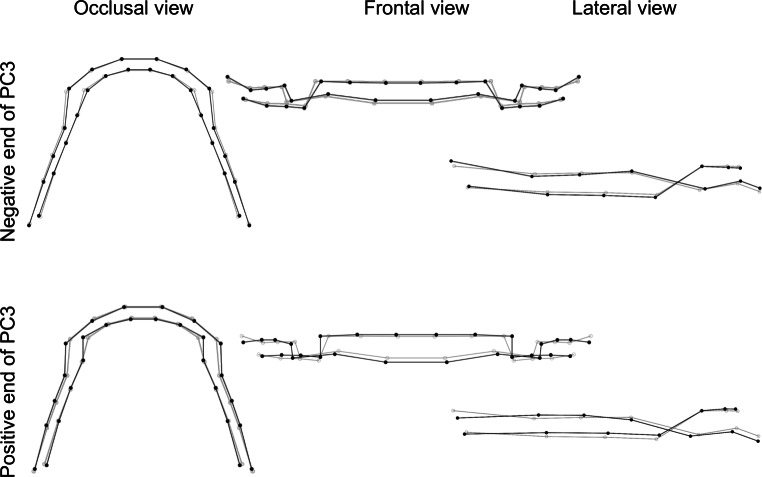
Visualization of dental arch shape changes in third principal component (PC3). The *gray* wireframe corresponds to the reference, consensus configuration Visualisierung von Veränderungen des Zahnbogens in der dritten Hauptkomponente (PC3). Das *graue* Netzgitter entspricht der Referenz, Konsenskonfiguration

The third principal component explained 11.6% of the variation in DAS in this sample. It varied from a DAS with a slightly decreased overbite to a slightly increased overbite. Anteroposteriorly, the wireframes representing the molar fossae curve ranged from concave to convex (Fig. 8).

### Partial least squares analysis

In order to analyze and quantify the relationship between the variables of this study, three PLS were performed (FS vs. DAS; FS vs. BF; DAS vs. BF). However, none of them showed a statistically significant result (Table 4). The covariation between the residuals of the sex regression of FS against DAS has a RV (coefficient that represents an overall measure of association between variables) value of 0.339, and a *p*-value of 0.689.

**Table 4:** Partial least squares analysis of covariation between FS and BF, and between DAS and BF PLS(„partial least squares“)-Analyse der Kovariation zwischen FS und BF sowie zwischen DAS und BF

Positions	FS, residuals from sex regression, RV value	FS, residuals from sex regression, *P*	DAS RV value	DAS *p*-value
Average MIC, right laterality, right MIC	0.138	0.668	0.224	0.184
Left MIC	0.192	0.292	0.118	0.612
Right MIC	0.134	0.594	0.166	0.342
Average MIC	0.154	0.474	0.160	0.362
Protrusion	0.150	0.510	0.061	0.961
Right laterality	0.118	0.725	0.240	0.115
Left laterality	0.053	0.992	0.149	0.419
Average MIC, left and right laterality, protrusion	0.154	0.919	0.268	0.217

*FS* facial shape, *BF* bite force, *DAS* dental arch shape, *MIC* maximum intercuspation

## Discussion

This study aimed to analyze the covariation between facial shape (FS), dental arch shape (DAS), and bite force (BF) by GM. Our null hypothesis that there is no correlation between these variables was confirmed after PLS analyses.

For shape, there was no statistically significant difference between DAS in men and women, but there was a significant difference for FS. In this sample, sexual dimorphism in FS was demonstrated using Procrustes ANOVA and DAF. Regarding the discrepancy between FS and DAS dimorphism, our results are in agreement with those of Alarcón et al. [31], who found in Spanish young adults, using GM, that there were sex-associated mandibular features that behave differently according to the facial pattern of the individuals. The sample used by these authors was similar to ours, both in terms of age range and in geographical location (Europe), but with the advantage of a larger sample size. However, contrary to our results, González et al. [40], also using GM methods, found a low craniofacial sexual dimorphism, with considerable overlap between female and male individuals in a PCA of 125 human skulls from the early 19th–20th centuries of known sex, aged from 15 to over 50 years old, from the Coimbra collection in Portugal. Although the sample was also European, it differed from ours in time period and age as well as in the nature of the data, since ours includes living individuals, and not skeletal remains. Similar results were obtained in a GM-based analysis of 20 skulls of contemporary Chilean adults [2]. The different conclusions reached by the various authors suggest that sexual dimorphism is not a prominent feature in modern populations, as it is in many nonhuman primates and mammals. The difference between male and female FS found in our study could be explained by sexual dimorphism, allometry, or sampling. Allometry is the effect of size on shape among individuals, where larger individuals should not be considered as scaled versions of the smaller individuals [41]. Allometry has been shown to influence sex differences in the shape of the human craniofacial complex, albeit in a two-dimensional geometric morphometric analysis [42].

Regarding DAS and sexual dimorphism, it has been observed that the size and dimorphic characteristics of dental arches and teeth decreased during human evolution [43, 44]. The causes of decreasing sexual dimorphism may involve different mechanisms. For example, Larsen [45] noted that primates with strong sexual dimorphism tended to have high levels of competition and confrontation between males (sexual selection). Although beyond the scope of this study, this idea would imply that during the evolution of the genus *Homo*, they have learned to live together without the need to physically confront each other, thereby diminishing certain features that allowed them to stand out from their peers. On the other hand, it has been proposed that the development of the lithic industry and, thus, extraoral food processing, and changes in the diet of modern humans are also factors that reduce sexual dimorphism in dental arches [43]. Perhaps linked to the latter, we did not find a statistically significant difference between sexes regarding BF. This result is in agreement with Abu Alhaija et al. [17] and Farias et al. [20]. However, several other studies have reported that men have higher BF than women [16, 19, 50, 51]. Therefore, our results in this regard could also be linked to sampling. The effect of the change in dietary consistency during human evolution is still not fully understood. Although males, in theory, may potentially exert larger muscle forces, it does not necessarily mean that they do. Self-awareness might unconsciously limit the exerted maximal muscle force, as this is normally not required for feeding. The complexity in describing the effect of a soft diet may be the reason for the observed differences in reported morphological and functional parameters. For example, the effect could differ for males and females, reducing male–female differences in functional parameters. Moreover, not fully reaching functional potentials could also be a factor that increases the overall human FS variation, as discussed below.

In dentistry, it is a common notion that dolichofacial individuals have a “long and narrow” face, whereas brachyfacial individuals have a “short and wide” face, and mesofacial individuals have a similar facial length and width. These conclusions are mainly based on linear morphometric studies, and a preliminary check (not shown) revealed a rather heterogeneous relationship between facial length and width in our sample. Therefore, it was rather unexpected that our PCA results showed that the main variation in FS was observed on the sagittal plane and not the frontal plane, specifically in the anterior and posterior facial height, and the resulting mandibular rotation. However, this aspect could be extrapolated to the aforementioned facial morphotypes, where a dolichofacial individual has a posterior mandibular rotation and a brachyfacial individual has an anterior mandibular rotation. There are some mechanical implications of the observed mode of shape variation, as a more posterior mandibular rotation tilts the force arm of the mandible and, thus, reduces the BF. However, in our sample these characteristics did not covary with the magnitude of BF. The relationship between BF and FS has been widely documented, and most studies claim that broad faces have the highest rank of BF, whereas long faces have the lowest [16, 17, 52]. Nonetheless, a lack of relationship between these variables has also been described [53]. These different findings suggest that mechanics may not be the main underlying factor of the differences between human facial biotypes.

The same is true for the shape variation of the dental arches, which went from a parabolic to an ovoid shape. This pattern of variation was similar to previous reports using linear morphometrics [54, 55]. Yet, the covariation analysis showed that, as with FS, there was no relationship with BF. Moreover, FS and DAS did not covary either. Paranhos et al. [9] investigated the relationship between the mandibular arch morphology and the facial type using lateral radiographs and dental casts and found no relevant relationship between these variables. However, other authors concluded that there is a direct relationship between facial and dental arch morphology, long faces having narrow dental arches and short face having wide arches [6, 8]. However, these studies used 2D linear morphometrics, which may have obscure shape changes, having size, rather than shape, as the main variable.

To put it in perspective, our results and those of the studies cited here (and many more [2, 3, 11, 39, 56]) do not necessarily reflect a lack of relationship, but a perhaps weak one in the age range presented here, or in the population from which our sample originates. However, it is interesting that modern humans lack a morphological pattern that reflects functional features of the human masticatory apparatus, suggesting a relative “disconnect” between form and function. This is not trivial since several clinical evaluations and decisions are based on this assumed relationship which is true for some mammals such as mice [57], wild carnivores [58], and others. However, this relationship appears to be more complex in humans. As mentioned above, during human evolution, the intensity of the chewing load has decreased due to changes in diet [11, 39, 59]. Nowadays, food is highly processed by mechanical and chemical processes, which allows the use of low chewing force during feeding. It has been suggested that the decrease of chewing load reduces the functional constraints in cranial development, allowing a greater range of morphological variation and also causing different parts of the skull to respond independently to chewing loads [3, 39]. Regarding the maxilla and mandible, von Cramon-Taubadel [60] postulated that the mandible is more affected by masticatory forces than the maxilla. Within the cranium, Eyquem et al. [39], showed that the shape of the maxilla is more affected than the rest of the craniofacial skeleton. Nevertheless, this effect is not deterministic, and the emergence of other factors may be more relevant than reduced masticatory forces to increase morphological variation. Considering the results of a study on the mandibles of contemporary and archeological populations, using different populations and 2D and 3D geometric morphometrics, Toro-Ibacache et al. [11, 29] suggested that in the absence of mechanical masticatory constraint, other factors may have a more relevant effect on jaw shape variation. For example, altered cranial functions, diet, environment, and genetics have an important effect on the variation of mandibular morphology. These multiple potential sources of variation could, thus, increase morphological variation independently of masticatory forces, explaining the weak relationship between the facial shapes, the dental arches, and bite force in this study.

## Conclusion

Using three-dimensional (3D) geometric morphometrics on morphological and functional data, this pilot study supports the conclusion of previous studies with different 3D-based methodologies that there is a weak relationship between bite force variation and dental arch and facial shape. Considering the quality of data information provided by geometric morphometric analyses, it is recommended to consider this methodology as the one to use when aiming to test further the question driving this study, but bearing in mind that considering a low effect size for sample size calculation might be necessary.

## Data Availability

The data that support the findings of this study are available from the authors, but ethical restrictions apply to the availability of these data due to its origin (living individuals who consented to participate in this study). Derived numerical data are, however, available from the authors upon reasonable request and with permission from the Ethics Committee of the Faculty of Medicine, University of Leipzig.
